# Directional compass preference for landing in water birds

**DOI:** 10.1186/1742-9994-10-38

**Published:** 2013-07-08

**Authors:** Vlastimil Hart, Erich Pascal Malkemper, Tomáš Kušta, Sabine Begall, Petra Nováková, Vladimír Hanzal, Lukáš Pleskač, Miloš Ježek, Richard Policht, Václav Husinec, Jaroslav Červený, Hynek Burda

**Affiliations:** 1 Department of Game Management and Wildlife Biology, Faculty of Forestry and Wood Sciences, Czech University of Life Sciences, 16521 Praha 6, Czech Republic; 2 Department of General Zoology, Faculty of Biology, University of Duisburg-Essen, 45117 Essen, Germany

**Keywords:** Bird flight, Water birds, Magnetoreception, Magnetic orientation, Sun compass, Flight control

## Abstract

**Introduction:**

Landing flight in birds is demanding on visual control of velocity, distance to target, and slope of descent. Birds flying in flocks must also keep a common course of landing in order to avoid collisions. Whereas the wind direction may provide a cue for landing, the nature of the landing direction indicator under windless conditions has been unknown. We recorded and analysed landing directions of 3,338 flocks in 14 species of water birds in eight countries.

**Results:**

We show that the preferred landing direction, independently of the direction from which the birds have arrived, is along the north-south axis. We analysed the effect of the time of the year, time of the day (and thus sun position), weather (sunny versus overcast), light breeze, locality, latitude, and magnetic declination in 2,431 flocks of mallards (*Anas platyrhynchos*) and found no systematic effect of these factors upon the preferred direction of landing. We found that magnetic North was a better predictor for landing direction than geographic North.

**Conclusions:**

In absence of any other common denominator determining the landing direction, the alignment with the magnetic field lines seems to be the most plausible if not the only explanation for the directional landing preference under windless and overcast conditions and we suggest that the magnetic field thus provides a landing direction indicator.

## Introduction

Landing is the most challenging and complex part of flight in both, aircraft and birds. Birds must visually control velocity, distance to the target and slope of descent. Birds flying in flocks must also coordinate their landing flight in order to avoid collisions with their neighbours. This is of particular importance in larger water birds (such as geese and ducks) which have longer braking distances when landing on water surface, when manoeuvring possibilities are constrained. The birds may synchronize the landing direction in that they all copy the direction of the leading bird. Not all flocks have, however, a defined leader, and the birds land often simultaneously, in an extended formation, rather than sequentially, in a row (own observations). Furthermore, every bird must strictly keep the common course of landing and must not deviate from it and cross the landing trajectory of any other bird. Whereas the landing course of an aircraft is dictated by air-traffic controllers and the "landing direction indicators" on airports, the birds have to "agree upon" a common directional cue. It should be noted that water birds often circle above the water before landing (probably as a mean of "security control"), and the landing direction usually does not correspond to the direction from which the birds have arrived. A reliable landing direction indicator is the wind and landing against the wind is also aerodynamically important. The problem is, however, which cue could serve as a landing direction indicator if there is no wind or only a gentle breeze. The position of the sun might be theoretically also used as a landing direction indicator, yet this cue is not always available. Given that birds use magnetic compass orientation and navigation for keeping the migration course and for homing [1-3] and given the fact that magnetic field provides a reliable, globally and at all times available orientation cue, we suggest that the magnetic field lines may serve also as a landing direction indicator. To test this hypothesis we measured the direction of landing of birds on water surface. The question was, whether under windless conditions the direction of landing is random or whether a certain compass direction of landing is preferred as predicted by the "landing direction magnetic indicator hypothesis".

## Results

**Figure 1:**
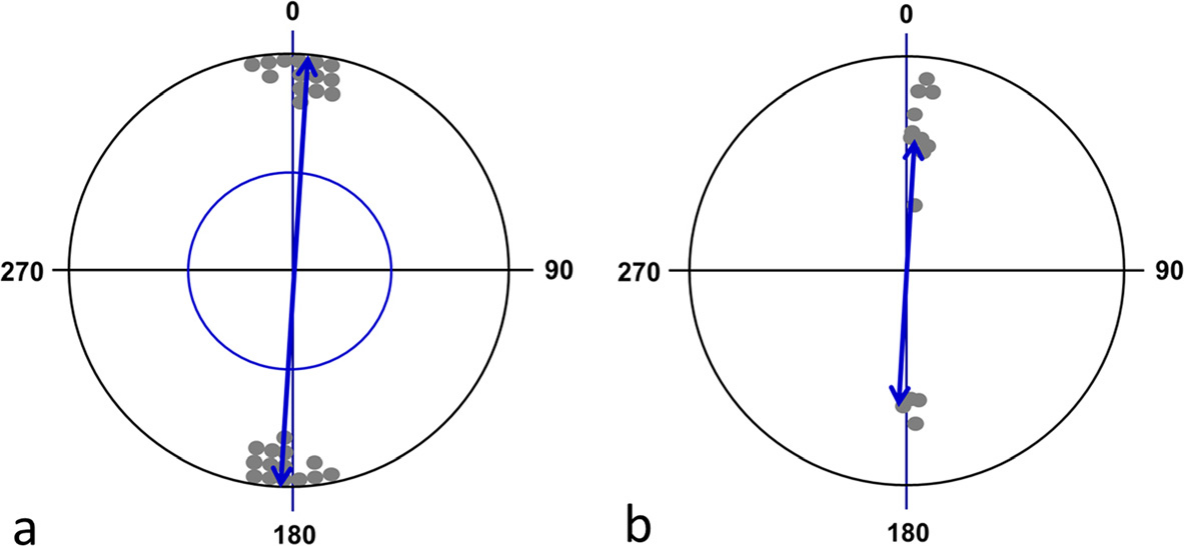
**Circular diagrams of the mean headings of landing water birds of all species investigated demonstrating the preference for landings along the north-south axis. (a)** Raw data plot. Each pair of dots (located on opposite sites within the unit circle) represents the direction of the second order bimodal mean vector calculated from the animals’ directional headings of one species (see Methods). The double-headed arrow indicates the grand mean axial vector calculated over all species. The inner circle marks the 5% significance level of the Rayleigh test. **(b)** Scatter plot summarizing statistics weighted by the length of the mean vectors of different species. The position of each pair of dots within the circle represents both the direction and the length of the bimodal mean vector of one species. The double-headed arrow indicates the weighted grand mean axial vector calculated over all species means.

Thirteen of the observed 14 bird species showed bimodally distributed landing directions which were significantly different from random distribution (Table 1). The grand mean vector of all species was highly significant and corresponded well with the geomagnetic north-south axis (μ = 3°/183° ± 3° (mean vector orientation angle; 95% confidence interval), r = 0.986 (mean vector length), Rayleigh test: n = 14, p = 8.36 × 10^-7^, Z = 13.616; second order (weighted) statistics: WMV: 3°/183°, r = 0.649, Hotelling test: n = 14, p = 1.82 × 10^-5^, F = 31.005; Figure 1).

**Table 1:** Axial headings of landing in 14 species of birds

**Species**	**n**	**mμ (axial)**	**Circular SD**	**r**	**p**
*Alopochen aegyptiacus*	189	175°/355°	28°	0.631	< 0.01
*Anas penelope*	91	7°/187°	16°	0.858	< 0.01
*Anas platyrhynchos*	2431	9°/189°	30°	0.575	< 0.01
*Anser anser*	16	6°/186°	29°	0.605	0.50 > p > 0.10
*Aythya ferina*	155	8°/188°	31°	0.558	< 0.01
*Cygnus olor*	15	3°/183°	28°	0.616	< 0.01
*Dendrocygna viduata*	30	4°/184°	28°	0.625	< 0.01
*Larus canus*	107	6°/186°	14°	0.885	< 0.01
*Larus ridibundus*	119	19°/199°	44°	0.301	< 0.01
*Mergellus albellus*	33	3°/183°	22°	0.735	< 0.01
*Mergus merganser*	22	5°/185°	17°	0.846	< 0.01
*Nettapus auritus*	14	176°/356°	23°	0.723	< 0.01
*Vanellus armatus*	31	177°/357°	29°	0.608	< 0.01
*Vanellus coronatus*	85	179°/359°	27°	0.643	< 0.01

n refers to the number of recordings (flocks), mμ: mean vector with respect to magnetic North, r: vector length, p: probability value of Rayleigh statistics.

### Flight phases

**Figure 2:**
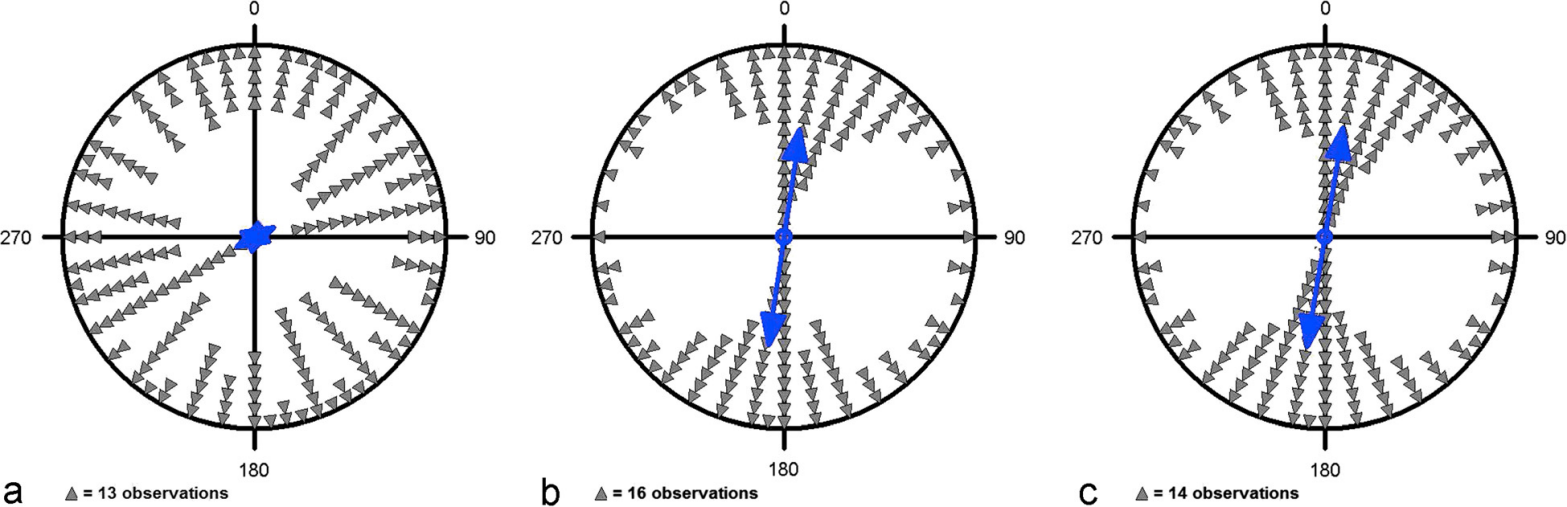
**Circular diagrams of the headings of mallards during different stages of the landing approach: (a) arrival (b) preparation (c) landing.** The mean vector of each phase is indicated by a double-headed arrow. Each triangle in **(a)**, **(b)**, **(c)** represents 21, 31, and 27 single measurements, respectively. The inner circle indicates the 5% significance level of the Rayleigh test.

While during arrival the mallards showed at best a slight preference for a southeast-northwest direction, during the preparation and landing phases they clearly preferred the north-south axis. The preference for north-south was highly significant for both of the last phases but the vector length r was increased in the final landing stage (arrival: μ = 62°/242° ± 7°, r = 0.11, n = 2,393, p < 10^-12^, Z = 29.206; preparation: μ = 8°/188° ± 1°, r = 0.567, n = 2,285, p < 10^-12^, Z = 733.848; landing: μ = 9°/189° ± 1°, r = 0.575, N = 2431, p < 10^-12^, Z = 802.861; Figure 2).

### Flock size

Even though just single data points were recorded for each flock independent of its size, a possible influence of the size of the flocks on landing direction was analysed. The mean flock size of mallards was 5 ± 7 (SD). The significant north-south preference during preparation and landing was found to be independent of flock size, but the circular standard deviation of the headings during landing decreased with increasing flock size (Pearson correlation, R^2^ = 0.961, p = 0.002). The accuracy of the landings was thus found to be highest in larger flocks compared to singly or pairwise flying birds.

Flock size in autumn (September-November) was significantly larger than in spring (March-May; Shapiro-Wilk normality test: p < 0.05; Mann-Whitney U-test, mean autumn: 8.6 ± 8.9, mean spring: 1.8 ± 1.5; p < 0.001).

Interestingly, a positive correlation between the mallard flock size and the eastward deflection of the mean landing direction from the magnetic north-south axis was also revealed (Pearson correlation, R^2^ = 0.952, p = 0.003).

### Countries, localities, and observers

**Figure 3:**
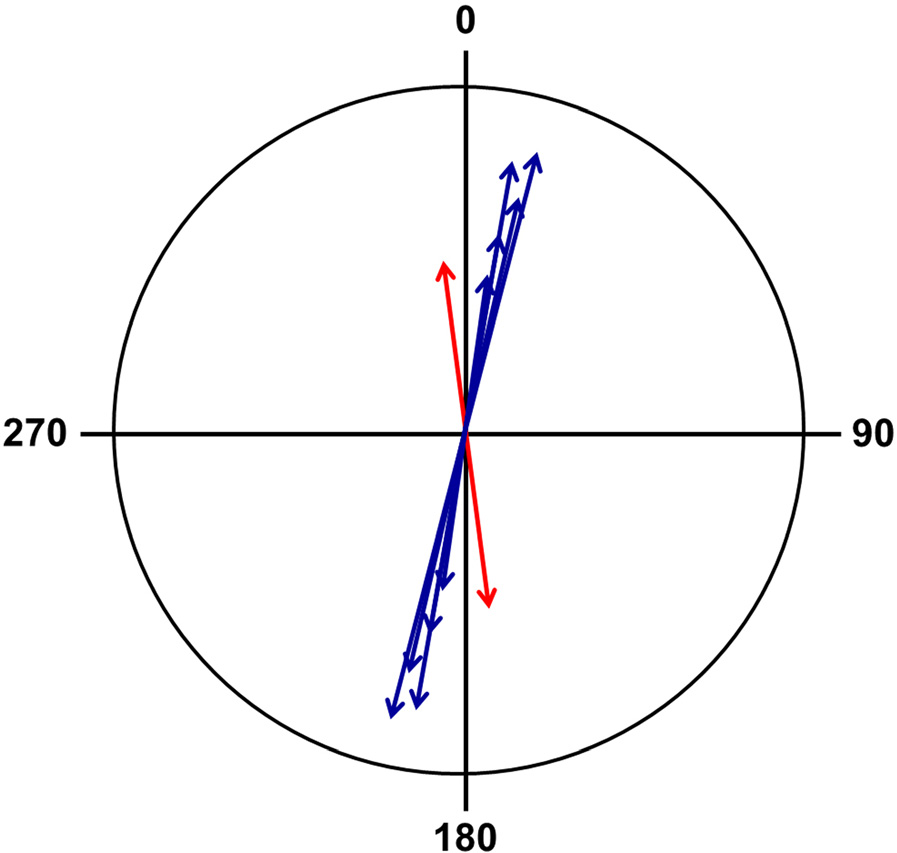
**Circular diagram of the weighted mean headings of landing mallards observed in different countries.** Populations from the eastern hemisphere (blue arrows) show NE-SW axis preferences while the only population from the western hemisphere (Canada, red arrow) exhibited a NW-SE landing preference.

No systematic differences were found between the headings of landing mallards at the different localities as well as between the datasets of different observers. However, there were significant differences between the data sets of the different countries regarding mean vectors as well as distributions even when sample size was normalized (multisample Watson-Williams F-test; F = 7.238, p = 1.13 × 10^-7^; multisample Mardia-Watson-Wheeler test, W = 292.709, p < 10^-12^). Interestingly, while the mallards in all countries of the eastern hemisphere preferred a NE-SW landing axis, the only group from the western hemisphere (Nanaimo, Canada) showed a deflection towards the NW-SE axis (Figure 3). The mean direction of the landings in western Canada was significantly different from the mean landing directions in all other countries (p < 0.01; pairwise Watson-Williams F-test, Table 2).

### Wind

As expected, wind was found to influence the landing directions but the significant preference for the north-south axis was retained under all wind directions. (Note, however that we recorded only under breeze not under stronger wind conditions). A weak positive correlation between the wind directions and the angular directions of landing indicated the birds preference for landing against the wind (circular-circular correlation coefficient, n = 736, r = 0.006, p < 0.05). However, the distribution of the mallard angular landing directions was highly significantly different from the distribution of wind directions (Mardia-Watson-Wheeler test: W = 30.483, p = 2.4 × 10^-7^). We can conclude that direction of breeze had no major influence on the landing directions.

### Sun position

**Figure 4:**
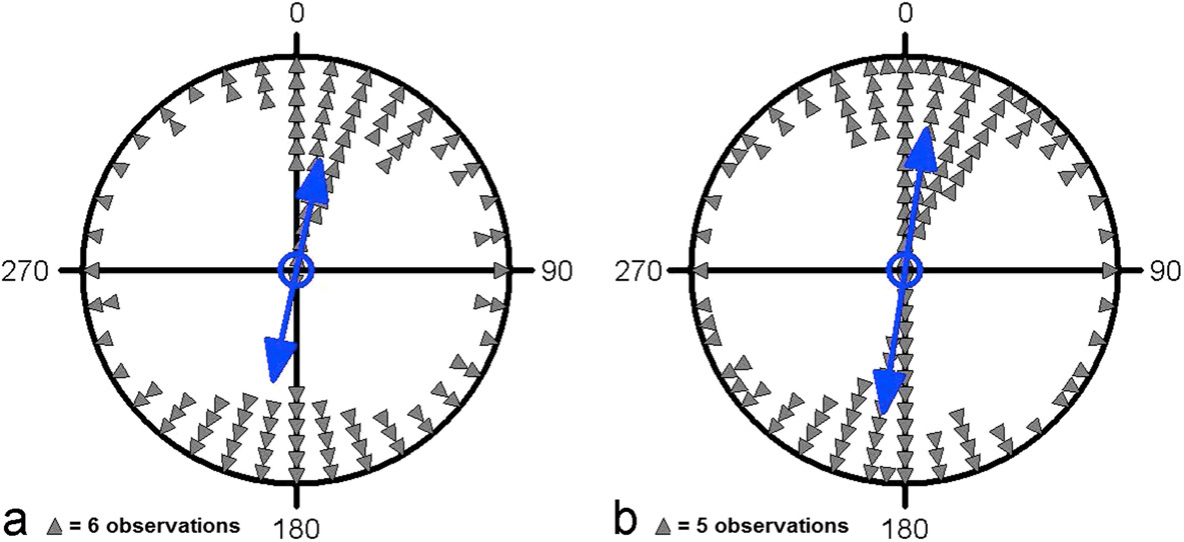
**Circular diagrams of the headings of landing mallards (n = 554 for each group) under different weather conditions: (a) clear sky (b) overcast.** The mean vector in each condition is indicated by the double-headed arrow. Each triangle in **(a)** and **(b)** represents 7 and 9 measurements, respectively. The inner circle indicates the 5% significance level of the Rayleigh test. The same results were obtained when we used the whole sample; the balanced subsamples with a sample size of n = 554 were chosen for better illustration.

Statistical differences were found between the landing directions on cloudy (overcast) vs. sunny days with less scattered data under overcast conditions (n = 554 for each group; Mardia-Watson-Wheeler test; W = 40.825, p = 1.36 × 10^-9^; Figure 4). The position of the sun on sunny days was weakly correlated to the direction of landing (circular-circular correlation coefficient r = 0.012, p < 0.05). However, the distribution of the mallard angular landing directions differed significantly from the distribution of sun positions (Mardia-Watson-Wheeler test: W = 383.436, p < 10^-12^). Thus, we conclude that sun position had just minor influence on landing directions.

### Time of day

The landing directions of the mallards were significantly different between midday (10 am till 3 pm) and all other times of the day both with respect to the mean vector as well as the distribution (n = 282 for each group; multisample Watson-Williams F-test; F = 8.215, p = 2.07 × 10^-5^; multisample Mardia-Watson-Wheeler test, W = 59.803, p = 4.93 × 10^-11^). Landings during midday showed a higher deflection towards east and had a larger r-value as well as a smaller circular standard deviation (n = 282 for each group; midday: μ = 16° ± 3°, r = 0.8; mean of all other daytimes: μ = 7° ± 4°, r = 0.552).

### Season

**Figure 5:**
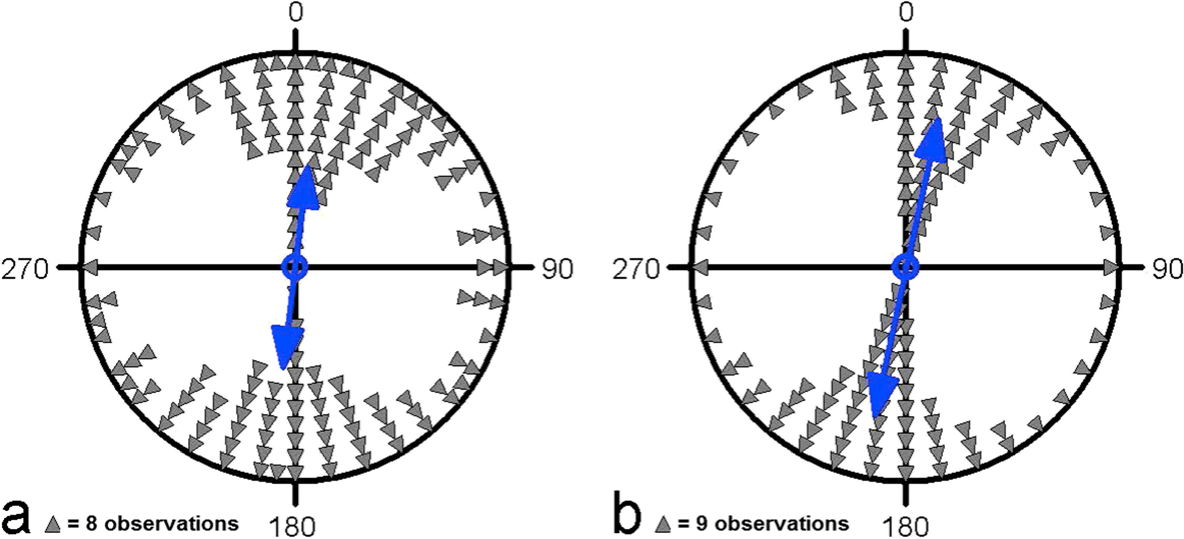
**Circular diagrams of the headings of landing mallards (n = 1,040 for each group) during different seasons: (a) spring (b) autumn.** The mean vector of each season is indicated by the double-headed arrow. Each triangle in **(a)** and **(b)** represents 12 and 16 measurements, respectively. The inner circle indicates the 5% significance level of the Rayleigh test. The same results were obtained when we used the whole sample; balanced subsamples with a sample size of n = 1,040 were chosen for better illustration.

Landing directions did also differ between the different seasons of the year (multisample Watson-Williams F-test; F = 12.743, p = 2.91 × 10^-8^; multisample Mardia-Watson-Wheeler test, W = 106.287, p < 10^-12^). The majority of measurements was conducted in spring (March – May) and autumn (September – November). Landing directions were less scattered and on average more deflected to east in autumn compared to spring (n = 1,040 for each group; spring: μ = 7°/187° ± 2°, r = 0.467; autumn: μ = 12°/192° ± 1°, r = 0.702; Watson-Williams F-test; F = 15.666, p = 7.81 × 10^-5^; Mardia-Watson-Wheeler test, W = 225.377, p < 10^-12^; Figure 5).

### Magnetic declination

The magnetic declination (angular difference between magnetic North and geographic North) at the studied localities varied between 3°-4° (Czech Rep., Italy, Poland) to 15°-17° (in western Canada and southern Botswana). The difference between the mean heading (mμ) of the water birds and magnetic North (mN) was significantly smaller than the difference between the mean heading (gμ) and geographic North (gN) calculated for each country separately (one-tailed paired t-test, ∣delta (mN, mμ)∣: 8.3° ± 4.3°; ∣delta (gN, gμ)∣: 14.9° ± 3.7°; p < 0.001, Table 2). Delta refers to the smallest angle between mN and mμ (and gN and gμ, respectively).

**Table 2:** Magnetic North versus geographic North as reference for the preferred heading direction (μ) during landing on water surface in the mallard (or diverse other species of water birds in the case of Botswana)

**Country**	**Magnetic declination**	**Number of flocks**	**Mean vector (mN) mμ**	**Mean vector (gN) gμ**	**∣Delta (mN, mμ)∣**	**∣Delta (gN, gμ)∣**
Botswana I	-8°	264	356°/176°	344°/164°	4°	12°
Botswana II	-15°	85	0°/180°	345°/165°	0°	15°
Canada	17°	136	354°/174°	11°/191°	6°	11°
Czech Rep.	3°	1,095	7°/187°	10°/190°	7°	10°
Estonia	7°	168	14°/194°	21°/201°	14°	21°
Finland	8°	218	9°/189°	17°/197°	9°	17°
Italy	3°	394	9°/189°	12°/192°	9°	12°
Poland	4°	381	13°/193°	17°/197°	13°	17°

Mean magnetic declination (angular difference between geographic North and magnetic North) is given for each locality. gμ and mμ denote the axial mean vectors (given as XX°/XX°) with reference to magnetic North (mN) and geographic North (gN), respectively. All mean values are highly significant (Rayleigh test, p < 0.000000001, 0.462 < r < 0.848). Delta refers to the smallest angle between mN and mμ (and gN and gμ, respectively).

### Measurement accuracy

**Figure 6:**
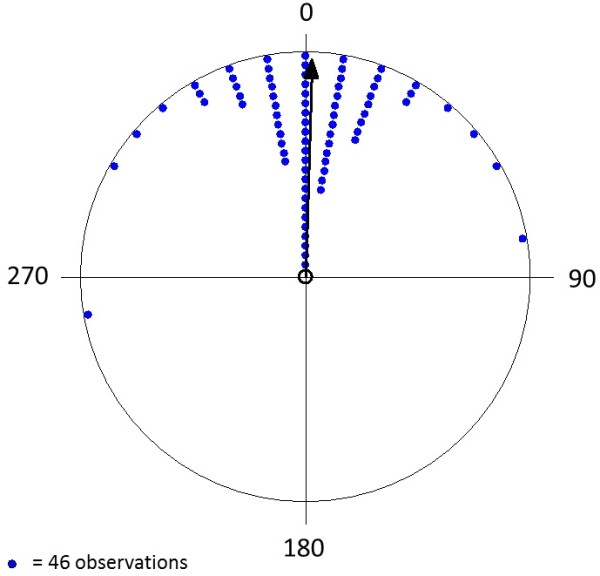
**Results of the test of accuracy of field measurements (i.e. estimation of direction) of eight observers.** The given angular values refer to difference between the real and the estimated values.

The difference between the assessed and actual directions (∆) was on average 1° (circular SD = 16°, r = 0.963, n = 3,184, p < 10^-12^, see Figure 6). (Note that although each of the eight persons should have made 400 trials, some of the observers did not catch the relatively high rate and missed some records). The performance (i.e. accuracy of recording) was highly uniform - and there were no significant differences between particular trials (i.e. observers did not get tired and less accurate, and they also did not get more accurate in the course of the experiment). There were also no significant differences in performance of particular observers, although the most experienced observers (Hart and Kušta, who also collected more than 50% of the data) showed an assessment accuracy with a smaller error (9.7° and 10.7° circular SD, respectively) than less experienced observers (the worst performance was characterized by ∆ = 0°, circular SD = 20°, r = 0.939, p < 10^-12^, n = 400; i.e. still highly accurate).

## Discussion

The recordings of landing water birds made by different observers, at different localities, at different times of the year and at different times of the day revealed a significant deviation from random distribution with a preference for landing along the approximate north-south axis. This phenomenon raises the following questions: 1) how reliable and how accurate are the field measurements, 2) what is the variance in the observed landing directional preference, 3) what are the cues determining the directional preference, 4) what is its function and adaptive meaning?

### 1. Accuracy and reliability of the field measurements

The assessment of the heading of subjects made by the observers at 100 m distance was surprisingly highly accurate and unbiased. The circular standard deviation (10°-15°) representing the recording (subjective) error should actually be subtracted from the observed circular standard deviation (about 30° in the mallard), so that we may assume that the real (objective) preference for landing direction was even more pronounced than our observation could have revealed. It should be stressed that the difference between the real heading of subjects and the observers’ estimation was zero for all observers. It should also be noted that the task to record movement direction of a person across 5 m at a distance of 100 m without binoculars is much more difficult than recording landing direction of birds on water surface using binoculars. Observing a duck even at a distance of 100 m with binoculars with magnification factor 10 is comparable to observing the duck at a distance of 10 m without binoculars. Since the field of view is narrower if looking through the binoculars, the observer can better focus upon the subject. Also the trail on the water left by the landing duck provides more time to the observer to make accurate directional estimation. All observers are field zoologists highly experienced in observing through binoculars and reading a compass.

### 2. Variance in the heading during landing

The "accuracy" of the landings (i.e. the least scatter around the mean vector) was the highest in larger flocks compared to singly or pairwise flying birds. This may be due to the fact that the landing direction reproduced by several birds can be better and more accurately recorded by the observer than a single landing event. Yet, this may be due also to the higher necessity of synchronizing the direction (and thus subordinating the own direction to the "prescribed" common one) in larger flocks in order to avoid collisions, or to correct own navigational errors across the group (the so-called "many wrongs principle", cf. [4]).

NNE-SSW deflection of the preferred (landing) axis from the “ideal” N-S axis, observed in Europe is a phenomenon which we encounter also in magnetic alignment of diverse other animal species in diverse behavioural contexts: in the grazing and resting cattle, red deer, and roe deer [5-8], hunting foxes [9] or schooling carps [10]. Interestingly, while the mallards in all countries of the eastern hemisphere preferred a NNE-SSW landing axis, the mallards from western Canada and water birds form southern Botswana, i.e. from regions with high absolute declination) showed a deflection towards the NW-SE axis. Since these data were recorded only by two single observers, we cannot exclude that the shift represents an observer's effect, although it is unlikely since one-sided biases did not occur in a single of the eight participants of our test of measurement accuracy. Furthermore it is of interest that the study of aerial images in Google Earth also revealed a NNW-SSE deflection in alignment of cattle in the western hemisphere [5,8]. Currently, we have no explanation for this phenomenon which surely deserves further examination.

Landing directions were less scattered around the mean in autumn compared to spring. While the mallards in spring were already breeding they were migrating or preparing to migrate in autumn. However, the autumn recordings also involved larger flocks than spring recordings, so that the observed difference in accuracy may be the effect of flock size (as discussed above) rather than that of the season.

### 3. Cues determining north-south heading

Under windless conditions the birds can orientate according to the actual sun position, by means of a sun compass, or using a magnetic compass. Many birds can keep a course relative to the sun and compensate for temporal changes of the sun's azimuth. Orientation according to this sun compass [cf. e.g. [11,12] has been demonstrated in the context of migratory navigation or homing. A sun compass requires more complex sensory and cognitive abilities than a simple magnetic compass, so that we consider alignment with the magnetic field lines to be a more suitable solution for a simple stereotypic non-goal oriented task like keeping a steady landing course. Of course, nature may follow other strategies than predicted by human logics. Three findings are, however, in favour of the magnetic compass: Firstly, the directional landing preference did not change throughout the year and between e.g. northern Italy and Finland, although the relative position of the sun was markedly different between these countries and between seasons. Secondly, under cloudy and foggy conditions, the north-south-landing preference was highly significant (and actually displayed a somewhat lower scatter than under sunny conditions, see Figure 4B) indicating that a putative magnetic compass plays an important role but may be overrun by orientation with respect to the sun if it is visible. Thirdly, we show that in all the studied localities, magnetic North was a better predictor for heading during landing than geographic North.

### 4. Why do the birds prefer a common landing direction?

While the preference for landing of water birds along the north-south compass axis is undisputable, the real challenge is to explain the biological significance of this orientation behaviour. We suggest that magnetoreception plays a role in the control and steering of synchronous landing in one direction, i.e. that the magnetic field direction provides a landing direction indicator as suggested in the introduction. The fact that magnetoreception has recently been shown in mallards, strengthens this hypothesis [13,14].

The existence of a common preferred direction (the so-called nonsense orientation) which is exhibited by birds (and among them particularly in the mallard) on release and which does not appear to be related to the migration or homing direction was reported earlier [15,16]). We agree with [16] that this common direction may facilitate flock formation in an escape context. It is apparent that a jointly preferred direction may not only enable flocking during take-off but also, and maybe more importantly, during landing. The fact that also single birds land in the jointly preferred compass direction does not falsify the hypothesis that the function of keeping the common direction is actually to avoid landing collisions. We may assume that any inborn directional preference will be kept instantly irrespective of the actual flock size.

Further, not necessarily alternative, yet in any case a speculative explanation is that retinal magnetoreception (sensu [17]) serves as an inclinometer to estimate and control the angle of descents. Changes in the vertical plane can be visually controlled using the angle between e.g. the tip of the bill and the horizon. However, since these two references lie in two different focal planes, they cannot be focussed simultaneously and their comparison is thus not optimal for the bird. If, as hypothesized, birds perceive the magnetic field as a pattern of visual modulation, they might learn which magnetically excited area on the retina corresponds to the optimal angle of descent, provided that the head is kept at a constant angle to the horizon (which may be realized by placing the image of the horizon in the area of highest visual acuity). Accordingly, the bird would just have to obtain a specific and constant relationship of the two excited areas on the retina to ensure an optimal touchdown. The usage of such a "retinal magnetic inclinometer" would thus explain why birds keep their heads relatively straight during landing (cf. [18]).

**Figure 7:**
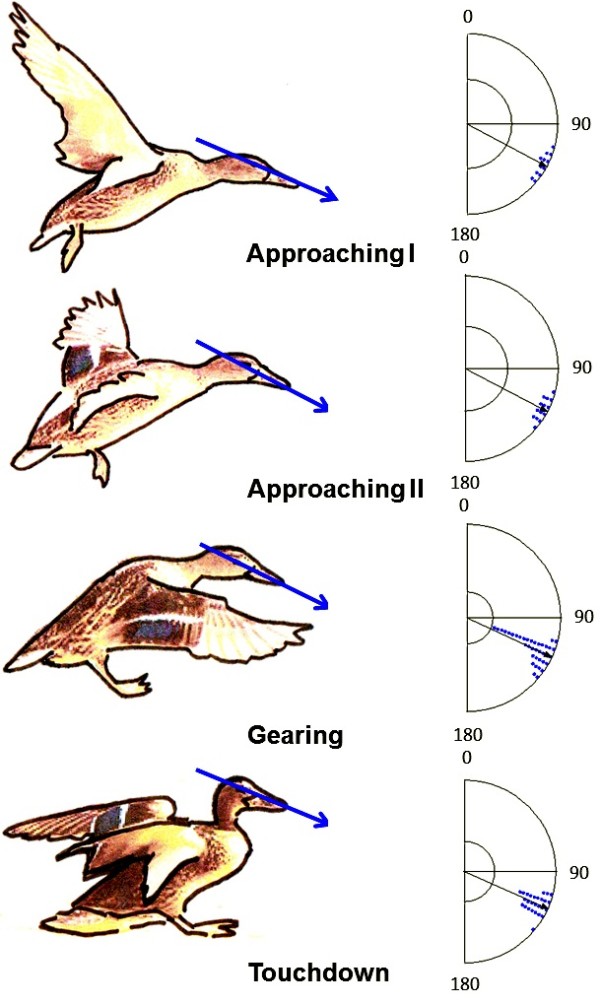
Head position of mallard ducks in the four phases of landing flight as estimated from photos available in internet.

Indeed, the head position in landing mallard ducks measured (as an angle between the line connecting eye and tip of the beak and the horizon = 90°) on photos available on the internet revealed a significantly uniform orientation (n = 91, μ = -25°, SD = 7°, r = 0.993, p < 10^-12^, see Figure 7 and Table 3).

**Table 3:** Head position of mallard ducks in the four phases of landing flight as estimated from photos available in the internet

	**Approach I**	**Approach II**	**Gearing**	**Touchdown**
Number of observations	12	14	38	27
Mean vector (μ)	118°	117°	115°	114°
Length of mean vector (r)	0.991	0.993	0.994	0.995
Circular standard deviation	8°	7°	6°	5°
Rayleigh test (p)	2.54 × 10^-11^	< 10^12^	7.9 × 10^-7^	1.67 × 10^-6^

The mean vector μ refers to the smallest angle between the line connecting the eye and the tip of the beak and the horizon (90°).

## Conclusions

Our study revealed that water birds landing on the water surface under windless conditions prefer to land roughly along the geomagnetic north-south axis, irrespective of the direction of arrival. In absence of any other common denominator determining the landing direction, alignment with the magnetic field lines seems to be the most plausible if not the only explanation for the directional landing preference, and we suggest that the magnetic field thus provides a "landing direction indicator". The preference of a common landing trajectory may be of significance to prevent collisions, particularly in larger birds with longer braking distances and which fly in pairs or flocks. Wind may be also preferred as a landing direction indicator because of aerodynamic reasons. Sun position (and sun compass as a course keeping mechanisms) cannot be excluded as a landing direction indicator if sun is visible, yet magnetic North is a better predictor of the landing direction than geographic North. Another hypothesis for the observed phenomenon assumes that in the preferred north-south direction birds can use radical-pair-based magnetoreceptors located in the retina to assess and control the slope (inclination) of landing. To test this hypothesis, recordings (using high-speed cameras) of the landing success, length of the braking trail, slope of landing, angle of the head etc. in localities with different inclination and magnetic field strength will be needed.

## Methods

The directions of 3,338 landing approaches of birds of 14 different species were measured at 30 different water bodies in eight countries by nine independent observers (Tables 4, 5). The vast majority of the landings occurred on standing water bodies; less than 3% of the data is related to landings on land or on rivers. Since more than 70% of the observations were done on the mallard (wild duck, *Anas platyrhynchos*, n = 2,431) this species was chosen for a detailed analysis. The mallard was observed at 25 localities in seven different countries by eight different observers (Table 4).

**Table 4:** List of localities and their magnetic field parameters

**Country**	**Locality**	**Latitude**	**Longitude**	**Inclination**	**Declination**	**Intensity (μT)**		
						**H**	**V**	**T**
Botswana	Chobe Kasane	17°49'17.14"S	25°07'51.12"E	-58	-7	16	-26	30
Botswana	Savuti I	18°36'12.89"S	24°04'20.27"E	-59	-8	15	-26	30
Botswana	Savuti II	18°39'16.90"S	24°03'51.71"E	-59	-8	15	-26	30
Botswana	Ngungungu	18°41'45.73"S	24°03'02.54"E	-59	-8	15	-26	30
Botswana	Gaborone	24°39'04.84"S	25°56'00.47"E	-63	-15	13	-25	28
Canada	Nanaimo	49°10'15.32"N	123°58'18.14"W	70	17	19	51	55
Czech Rep.	Kladno	50°07'41.82"N	14°08'17.08"E	66	3	20	45	49
Czech Rep.	Mohelnice	49°46'50.28"N	16°57'24.48"E	66	4	20	45	49
Czech Rep.	Kunratice-Seberak	50°00'40.49"N	14°29'34.77"E	66	3	20	45	49
Czech Rep.	Litomysl-Lucni	49°51'38.86"N	16°17'49.05"E	66	4	20	45	49
Czech Rep.	Kunovska tabule	49°01'23.88"N	17°25'49.18"E	65	4	20	44	49
Czech Rep.	Tezebni jezero	49°00'35.52"N	17°24'79.90"E	65	4	20	44	49
Czech Rep.	Tisov	49°30'44.60"N	13°49'34.06"E	65	3	20	44	49
Czech Rep.	Dobra vule	49°06'17.88"N	14°45'16.44"E	65	3	20	44	49
Czech Rep.	Skutek	49°06'35.84"N	14°45'10.09"E	65	3	20	44	49
Czech Rep.	Libaluv rybnik	49°10'39.71"N	14°41'01.64"E	65	3	20	44	49
Czech Rep.	Velky Honys	49°30'23.48"N	13°49'50.84"E	65	3	20	44	49
Czech Rep.	Bukovec	49°30'35.01"N	13°49'51.29"E	65	3	20	44	49
Czech Rep.	Podtisovsky	49°30'41.82"N	13°49'28.07"E	65	3	20	44	49
Czech Rep.	Mlynsky	49°31'09.94"N	13°48'54.17"E	65	3	20	44	49
Estonia	Männikuste	58°23'07.29"N	23°58'53.13"E	72	7	16	49	51
Finland	Vesijärvi	61°05'26.52"N	25°23'07.29"E	74	8	14	50	52
Germany	Bonn	50°42'46.36"N	07°08'41.57"E	66	1	20	44	49
Italy	Valle Cavanata	45°43'08.82"N	13°28'21.39"E	62	3	22	42	47
Poland	Milicz (4 Sites)	51°32'30.90"N	17°19'57.21"E	67	4	19	46	49

*H*, Horizontal; *V*, Vertical; *T*, Total magnetic field intensity. Values calculated by http://www.ngdc.noaa.gov/geomag-web/.

**Table 5:** Survey of sampling and the list of recorded species

**Species**	**N flocks**	**N specimens**	**Countries**	**N localities**	**N half-day sessions**	**Observer**
*Alopochen aegyptiacus*	189	189	RB	1	4	Hus
*Anas penelope*	91	646	FIN	1	1	Har, Kus
*Anas platyrhynchos*	2,431	12,235	CDN, CZ, D, I, EST, FIN, PL	25	70	Han, Har, Kus, Mal, Nov, Pat, Ple, Pol
*Anser anser*	16	26	D, PL	2	4	Mal, Har, Kus
*Aythya ferina*	155	200	PL	3	4	Har, Kus
*Cygnus olor*	15	19	EST, FIN, PL	3	3	Har, Kus
*Dendrocygna viduata*	30	932	RB	3	3	Hus
*Larus canus*	107	214	FIN	1	2	Har, Kus
*Larus ridibundus*	119	119	D, EST, PL,	3	4	Har, Kus, Mal
*Mergellus albellus*	33	68	FIN	1	2	Har, Kus
*Mergus merganser*	22	46	FIN	1	2	Har, Kus
*Nettapus auritus*	14	14	RB	1	4	Hus
*Vanellus armatus*	31	31	RB	2	1	Hus
*Vanellus coronatus*	85	85	RB	1	2	Hus
**Total: 14 species**	**3,338**	**14,824**	**8**	**30**	**78**	**9**

*N*, Number; *CDN*, Canada; *CZ*, Czech Republic; *D*, Germany; *EST*, Estonia; *FIN*, Finland; *I*, Italy; *PL*, Poland; *RB*, Botswana; *Han*, Hanzal; *Har*, Hart; *Hus*, Husinec; *Kus*, Kušta; *Mal*, Malkemper; *Nov*, Nováková; *Pat*, Patočková; *Ple*, Pleskač; *Pol*, Policht.

### Sampling

The flying directions (heading) of water birds prior to landing on a water surface were observed directly or by means of binoculars at a distance of 20-100 m. The compass directions were estimated to the nearest 5° by means of hand held compasses. We recorded compass directions (as corresponding to heading of the birds) at three different stages of the flight whenever possible: arrival, preparation for landing, and landing. Arrival was defined as the observed heading when the birds were first sighted. Preparation was defined as the compass direction during the last phase of the landing approach. The landing direction was recorded when touching the water surface.

In addition to the headings the following parameters were recorded for every observation: (1) species (2) flock size (3) weather conditions (4) time and date (5) direction of the observer towards the animals. It should be noted at this point that all the birds of a flock fly in synchrony - and the angular deviations of particular birds from the common direction are less than the recording error made by the observer. Therefore a single compass value was estimated for arrival, preparing, and landing of each flock regardless of its actual size.

Wind strength was measured with a digital hand held anemometer (Technoline EA 3000). The observations were made under conditions with wind strengths of maximal 5.4 m/s. Of all the recordings (n = 3,338), 70% were made under calm conditions (wind speed 0-0.2 m/s), 11% under light air (0.3-1.5 m/s), 9% under light breeze (1.6-3.3 m/s), 10% under gentle breeze (3.4-5.4 m/s).

### Control of the recording accuracy

To check the inter-observer reliability and repeatability of the assessment of heading directions we tested the measurement accuracy of observers. They were asked to determine the direction of movement of a person who walked along a straight line within a circle (radius 5 m) at a distance of 100 m to the observer. The observers did not use binoculars for this task. The person moved within each trial in 50 different exactly defined directions (always starting from the middle of the circle) following a randomized protocol that the observers did not know. The eight observers were distributed along the circle on eight cardinal positions and changed their positions after each trial, so that each observer made altogether 400 assessments (in eight trials from eight different positions). Eventually the assessments of the observers were compared with the real heading directions, and the angular deviations were calculated.

### Data analysis

Circular statistics were carried out with Oriana 4.01 (Kovach Computing). Mean vectors were calculated for the headings of birds at different localities, of different species, during different seasons, under different weather conditions, for different observers, and at different times of the day. Single data points were recorded for each flock of birds and these headings were used for the analysis. Doubling the angles was used to convert angular data in axial ones prior to statistical analysis.

First order (Rayleigh test) and second order (Hotelling test) statistics were employed to test the headings for significant deviations from random distribution. Possible correlations were investigated using circular-circular correlations or Pearson statistics (Microsoft Excel). We used the t-test to compare mean flock sizes at different seasons. Whenever sample sizes differed significantly between different conditions, they were normalized to n or subsamples were taken for comparisons. Differences between the mean headings and distributions between groups (species, localities, etc.) were tested for significance with the Watson-Williams F-test and Mardia-Watson-Wheeler test, respectively. Circular diagrams were plotted in Oriana 4.01.

### Ethics statement

The study did not involve any experimentation or disturbing animals under observation.

## Competing interests

The authors declare that they have no competing interests.

## Authors’ contributions

Design of the study: Bur, Har, Kuš, Nov. Data collection: Čer, Han, Har, Jež, Kuš, Mal, Nov, Ple, Pol. Data analysis: Beg, Bur, Har, Mal. Writing the paper: Beg, Bur, Har, Mal. All authors read and approved the final version of the manuscript.
